# In vitro and in vivo biological performance of hydroxyapatite from fish waste

**DOI:** 10.1007/s10856-021-06591-x

**Published:** 2021-08-28

**Authors:** João Paulo dos Santos Prado, Hirochi Yamamura, Angela Maria Paiva Magri, Pedro Luiz Muniz Ruiz, José Lucas dos Santos Prado, Ana Claudia Muniz Rennó, Daniel Araki Ribeiro, Renata Neves Granito

**Affiliations:** Department of Biosciences, Federal University of São Paulo, UNIFESP, Santos, SP Brazil

## Abstract

The aim of this study was to evaluate biocompatibility of hydroxyapatite (HAP) from fish waste using in vitro and in vivo assays. Fish samples (whitemouth croaker - *Micropogonias furnieri*) from the biowaste was used as HAP source. Pre-osteoblastic MC3T3-E1 cells were used in vitro study. In addition, bone defects were artificially created in rat calvaria and filled with HAP in vivo. The results demonstrated that HAP reduced cytotoxicity in pre-osteoblast cells after 3 and 6 days following HAP exposure. DNA concentration was lower in the HAP group after 6 days. Quantitative RT-PCR did not show any significant differences (*p* > 0.05) between groups. In vivo study revealed that bone defects filled with HAP pointed out moderate chronic inflammatory cells with slight proliferation of blood vessels after 7 and 15 days. Chronic inflammatory infiltrate was absent after 30 days of HAP exposure. There was also a decrease in the amount of biomaterial, being followed by newly formed bone tissue. All experimental groups also demonstrated strong RUNX-2 immoexpression in the granulation tissue as well as in cells in close contact with biomaterial. The number of osteoblasts inside the defect area was lower in the HAP group when compared to control group after 7 days post-implantation. Similarly, the osteoblast surface as well as the percentage of bone surface was higher in control group when compared with HAP group after 7 days post-implantation. Taken together, HAP from fish waste is a promising possibility that should be explored more carefully by tissue-engineering or biotechnology.

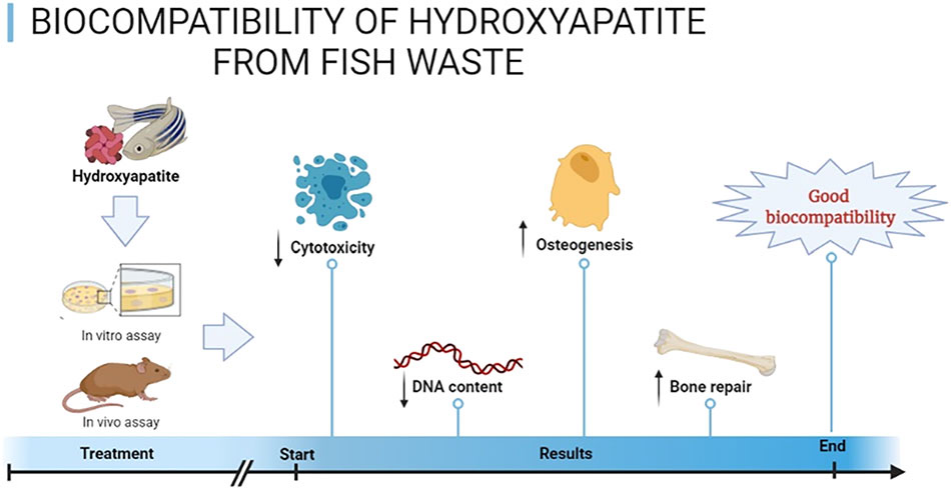

## Introduction

Hydroxyapatite (HAP) (represented by the chemical formula Ca_10_[PO_4_]_6_[OH_2_]) is a hexagonal-structured mineral belonging to the generic family of calcium phosphates called apatites [1]. It can be naturally found in mineralized tissues, such as bone, dental enamel and dentin, contributing for the notable biomechanical properties.

Bioactivity is the property of being biologically active and eliciting a response in the living tissues [2]. Of particular importance, Neo et al. [3] have demonstrated that HAP has bioactivity properties in bone tissue. In a study conducted by Lin et al. [4], pluripotent mouse stem cells have expressed some osteo-specific genes by HAP exposure. This is consistent with the idea that HAP-cell interaction occurs through osteoinductive potential able to stimulate the differentiation into osteoblasts [4].

Despite the positive features regarding synthetic materials, the high manufacturing costs is even an obstacle for clinical applications due to the possibility of controlling production, repeatability, reproducibility and traceability. In this context, many efforts have been made to optimize HAP production through different methods [5]. A growing number of studies have been conducted to extract HAP from different sources for bone regeneration [6–8]. Natural HAP is the most promising option for innovative source of developing, since it could represent an economically viable alternative for this project.

Fishes are considered to be an important food source for human health. This is due to the fact that fish contains a wide variety of compounds, which are highly beneficial health, such as protein, sodium, magnesium, potassium, calcium, and vitamins B6 and B12 [9]. However, it should be noted that many tons of fish scales are produced during fish processing around the world [10]. As a consequence, relevant amounts of raw materials are daily wasted and destined to landfill, namely human health concerns, and environmental consideration [11, 12]. Following this rationale, HAP may be extracted from skeleton of fishes, which were originally considered discards in the food market.

Some authors have successfully conducted scientific studies able to produce HAP powder from fish waste [13, 14]. In vitro investigation of cell-material interaction indicated good biocompatibility toward osteoblasts [13]. Subcutaneous implantation of HAP in rats showed a well-organized connective tissue after 30 days post-implantation, with slight or absent inflammatory response. These findings suggest that the use of the HAP from fish waste may be interesting for developers, as for example, tissue-engineering or biotechnology [14].

As a result, and because of positive scientific evidence, the aim of this study was to evaluate biological responses of HAP from fish waste by means of cytotoxicity, osteogenesis and bone repair at the cellular and molecular levels in vitro and in vivo.

## Materials and methods

### HAP powder synthesis

Fish samples (whitemouth croaker - *Micropogonias furnieri*) from the biowaste generated by the Seafood Market at the Santos city (Sao Paulo state, Brazil) was used as HAP source. The protocol for the synthesis of HAP powder was previously described by Yamamura et al. [14]. Briefly, HAP used in this study is based on the following physico-chemical properties: infrared spectroscopy analysis pointed out similar composition to HAP standard with the presence of carbonate ion demonstrated by wave number values of 871 cm^−1^ and 1420 cm^−1^ for calcinations at 800 °C. The scanning electronmicrographies depicted the crystal morphology and porous nature with average pore size of ~10 um. Plasma emission spectrometry Ca was 36.8; Mg was 0.8, Na was 0.7 and K was 0.5 [14].

This study was approved by Ethics Committee at Federal University of Sao Paulo, UNIFESP, Protocol number #231.533.

### In vitro study

#### Cell culture

Cytotoxicity of HAP powders was assessed to evaluate cell proliferation. First, HAP particles were sterilized using ultraviolet irradiation (UV) during 24 h. Then, HAP at the 0.05 g/mL concentration was incubated in α-MEM culture medium (alpha minimal essential medium with 10% fetal bovine serum and 1% antibiotic; Vitrocell, Campinas, Brazil) supplemented with 1% β-glycerophosphate, 1% 2-phospho-L-ascorbic acid trisodium salt, and 0.1% dexamethasone for 24 h in an incubator at 37 °C and 5% CO_2_. After that, the conditioned medium was collected and filtered using a 0.22-μm filter (Kasvi, Curitiba, Brazil). The control medium without the material was exposed to the same conditions as purposed by HAP samples.

Pre-osteoblastic MC3T3-E1 cells (BCRJ, RJ,Brazil) were used. Cell culture was performed in an incubator set at 37 °C and 5% CO_2_. When they reached 80% of confluence, the cells were detached using trypsin. Cells were then seeded in a 24-well plate in a density of 5 × 10^3^ cells/cm^2^ in 1 mL of standard culture medium. After 24 h, medium was replaced for 1 mL of the conditioned HAP medium (or standard culture medium in the control group). The cells were then incubated for the periods of 1, 3 and 6 days.

#### Alamar Blue^®^assay

Alamar Blue assay (Thermo Fisher Scientific, São Paulo, Brazil) was used to evaluate cytotoxicity of HAP on cell culture. For this assay, each well received 500 μL of 10% Alamar Blue solution being incubated for 3 h. After that, 200 μL of the solution was added into a 96-well plate. The 96-well plate was measured in the microplate spectrophotometer (Bio-Tek Instruments, Inc.) at 570 and 600 nm. The values obtained from the readings were used to calculate the Alamar Blue reduction rates according to the manufacturer’s instructions. The proliferation rate was obtained based on the Alamar Blue reduction rate values. The experiments were conducted in duplicate.

#### DNA quantification (Pico Green)

To evaluate the DNA quantification assay (QuantiFluor® dsDNA quantification kit; Promega, São Paulo, Brazil), all plates were washed twice with PBS and then, after two freeze-thaw cycles (−80 °C, 25 °C), 100 μL of fresh made working solution was added to each well. The plate was incubated for 5 min and the fluorescence signals (485/20 excitation and 528/20 emission) were read in a microplate spectrophotometer (Bio-Tek Instruments, Inc.).

#### Quantitative RT-PCR

The expression of Runx 2 and BMP4 genes were evaluated through qRT-PCR. For this assay, pre-osteoplastic MC3T3-E1 cells were seeded in 24-well plates (1 × 10^4^ cell/cm^2^) with conditioned or standard culture medium for the periods of 1, 3 and 6 days (*n* = 5). For each experimental period, total RNA was isolated using a RNA isolation kit (RNeasy Mini Kit, QIAGEN, São Paulo, Brazil). The RNase-free DNase I (Thermo Fisher Scientific, São Paulo, Brazil) kit was used to remove any potential contamination from the samples. After that, the complementary DNA (cDNA) was made from the current RNA using the RNA using the High-Capacity cDNA Reverse Transcription Kit (Thermo Fisher Scientific, São Paulo, Brazil). The qRT-PCR analysis was made on a thermal cycler (7500 Fast Real-Time PCR System, Applied Biosystems, Waltham, USA) with a SYBR green detection reagent. (Thermo Fisher Scientific, São Paulo, Brazil). The housekeeping gene RPS18 (ribosomal protein S18) was used to normalize the relative gene expression. Relative expression was calculated using the following formula: 2^-ΔΔCt.

### In vivo study

#### Surgical procedure and experimental groups

All animals were submitted to surgery for artificially creating critical bone defects in the rat calvaria as previously described by Cui et al. [15]. For this purpose, a circular, and full-thickness two bone defects (1.5 mm in diameter each) were created in the left or right side of rat calvaria. After that, HAP was implanted in the bone defects from the left side. Bone defects from right side were used as controls. The rats euthanized at 7, 15 and 30 days post-implantation.

#### Histological procedures

The skull samples were removed, fixed in 10% buffer formalin (Merck, Darmstadt, Germany) for 48 h. Decalcification was made using a 4% ethylenediaminetetraacetic acid (EDTA) (Merck, Darmstadt, Germany) for approximately 30 days. After that, the samples were embedded in paraffin blocks. 5 μm thin sections were obtained and stained with hematoxylin and eosin (Merck,Darmstadt, Germany). Sections were examined with a light microscopy (Leica Microsystems AG, Wetzlar, Germany, Darmstadt-Germany) for the presence of granulation tissue, inflammatory process, new bone formation and biomaterial removal. This analysis was performed in a blinded way per animal.

#### Histomorphometric analysis

Bone sections were also quantitatively analyzed with the OsteoMeasure System (Osteometrics, Atlanta, GA, USA). The region of interest (ROI) for the quantification of the structural bone parameters was defined as whole region between the two borders of the skull defect (ROI = 1.67 ± 0.48 mm^2^). The following quantitative parameters were obtained: bone volume as a percentage of tissue volume (BV/TV, %), osteoblast number per unit of tissue area (N.Ob/T.Ar, /mm^2^), osteoblastic surface as a percentage of bone surface (Ob.S/BS, %), biomaterial volume per unit of tissue area (BM.V/TV, %). The analysis was performed in a blinded way by one experienced observer (JPSP).

#### Immunohistochemistry

Immunohistochemistry was made according Ruiz et al. [16]. The specimens were incubated with anti RUNX-2 monoclonal primary antibody (Santa Cruz Biotechnology, USA) at a concentration of 1:200 followed by biotin conjugated secondary antibody anti-rabbit IgG (Vector Laboratories, Burlingame, CA, USA) at a concentration of 1:200. The conjugation was visualized by the application of a 0.05% solution of 3-3′-diaminobenzidine solution and counterstained with Harris hematoxylin.

### Statistical analysis

Data were expressed as mean and standard deviation. One-way analysis of Variance (ANOVA) was used, followed by Tukey’s multiple comparison post-test. *p* < 0.05 was considered as significant statistically. All statistical analyses were performed using Graph Pad Prism™, version 6.0.

## Results

### In vitro study

#### Alamar Blue

**Fig. 1:**
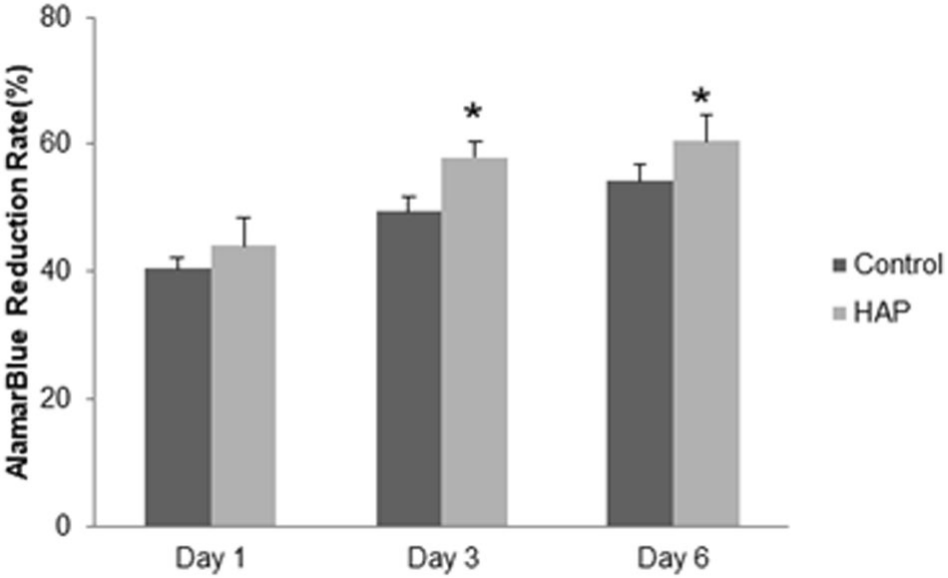
Alamar Blue reduction rate after 1, 3 and 6 days of MC3T3-E1 cell culture with standard (control) or HAP preconditioned medium. *p < 0.05 vs. control group (One-way ANOVA with Tukey’s test)

Cytotoxicity did not show significant statistically differences after 1 day of HAP exposure (*p* > 0.05) (Fig. 1). On the other hand, MC3T3-E1 cells exposed to the HAP after 3 and 6 days, showed that Alamar Blue reduction rate was higher in HAP samples when compared to control group (Fig. 1).

#### DNA quantification

**Fig. 2:**
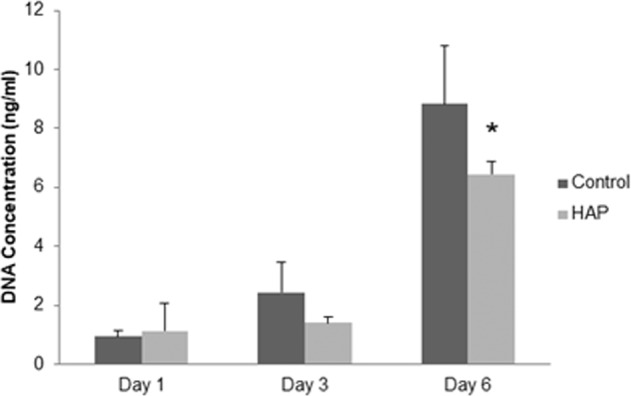
DNA concentration for control and HAP group after 1, 3 and 6 days of culture in standard and conditioned medium, respectively. *p < 0.05 vs. control group (One-way ANOVA with Tukey’s test)

DNA quantification by Pico Green failed to detect any significant differences (*p* > 0.05) between control and HAP groups after 1 and 3 days of exposure (Fig. 2). However, DNA concentration was lower in the HAP group after 6 days of exposure (Fig. 2).

#### Quantitative RT-PCR

**Fig. 3:**
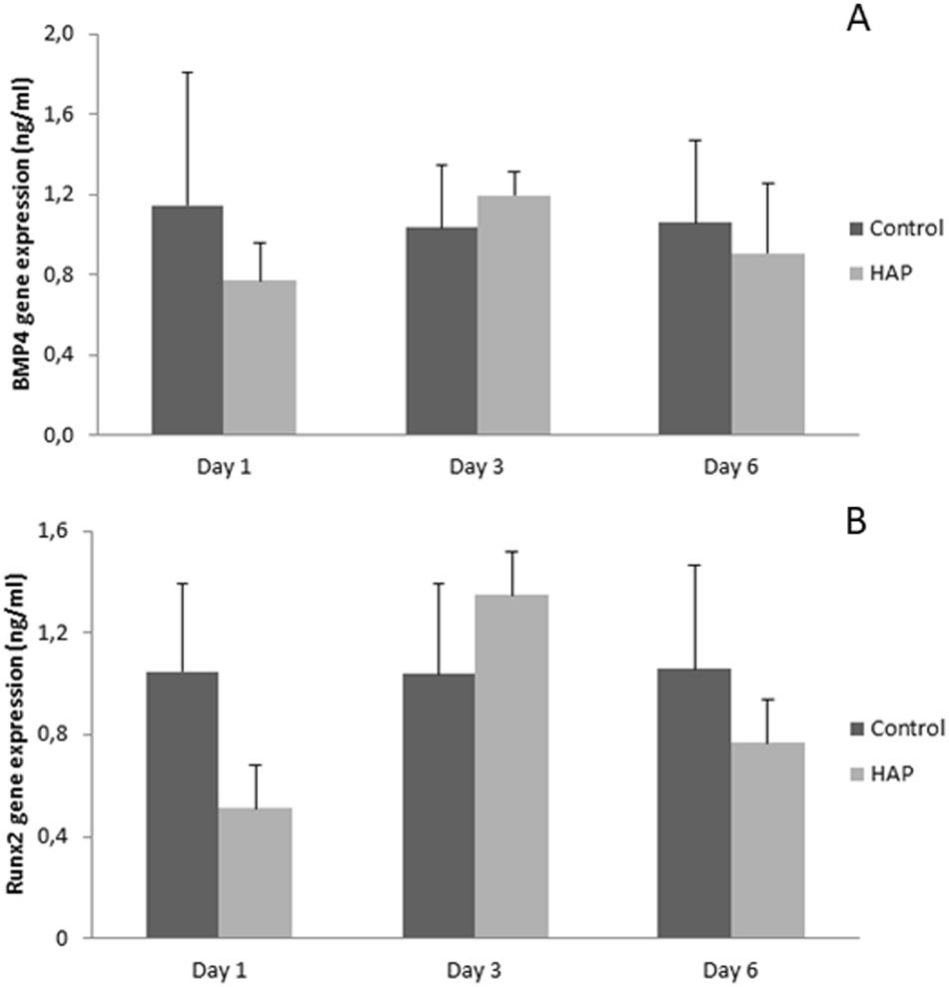
Quantitative RT-PCR for (**A**) BMP4 and (**B**) Runx2 genes in control and HAP groups after 1, 3 and 6 days of MC3T3-E1 culture in standard (control) and HAP-conditioned medium. *p* > 0.05

Quantitative RT-PCR showed that the preconditioned HAP medium did not alter the expression of BMP4 gene in MC3T3-E1 cells after 1, 3 and 6 days of exposure (Fig. 3A). Similarly, no significant statistically differences (*p* > 0.05) were observed between control and HAP groups for Runx-2 gene expression for all periods evaluated (Fig. 3B).

### In vivo study

#### Histopathological analysis

**Fig. 4:**
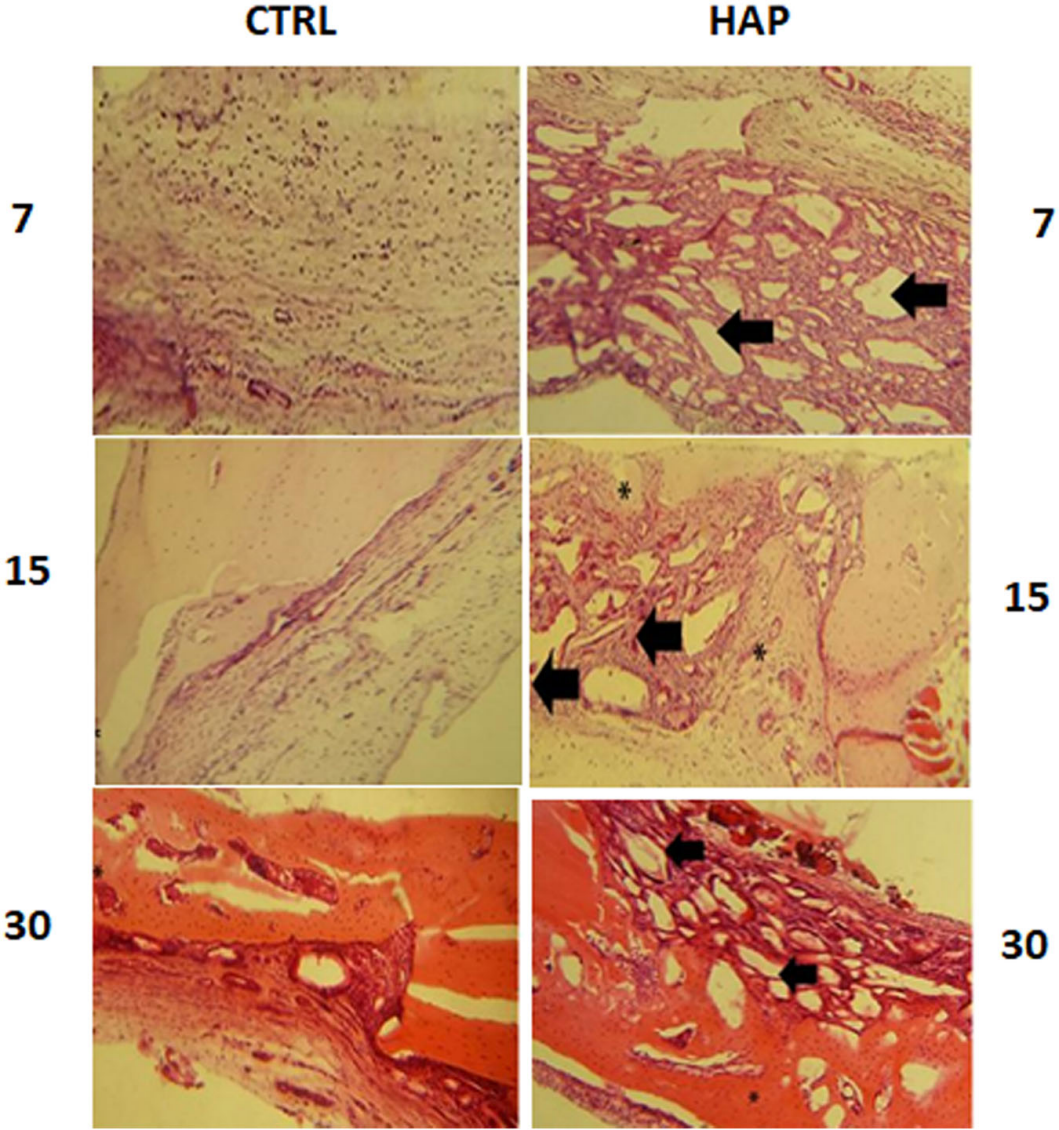
Photomicrographs of rat calvaria following HAP implantation after 7, 15 and 30 days post-implantation: CTRL control group, HAP hydroxyapatite group. Arrow: HAP; asterisk: bone formation H.,E. stain. ×40 magnification

In the control group, all bone defects showed self-repair within 30 days of surgery. This was confirmed by the presence of newly formed bone tissue inside the defect. However, the presence of granulation tissue was identified to all animals after 7 or 15 days of surgery (Fig. 4).

In the group treated with HAP, moderate chronic inflammatory cells depicted by lymphocytes, macrophages, multinucleated giant cells, and angiogenesis were observed after 7 days post-implantation. The presence of a great amount of HAP was also noticed in this group. In the bone defected filled with HAP for 15 days post-implantation, there was a decrease of chronic inflammatory infiltrate followed by considerable amount of HAP resorption. At the end of the experiment, chronic inflammatory infiltrate was absent for all animals investigated in this study. There was also a decrease in the amount of biomaterial associated with areas of recently formed bone tissue (Fig. 4). This pattern was very similar to that found in the control group.

#### Bone histomorphometry

**Fig. 5:**
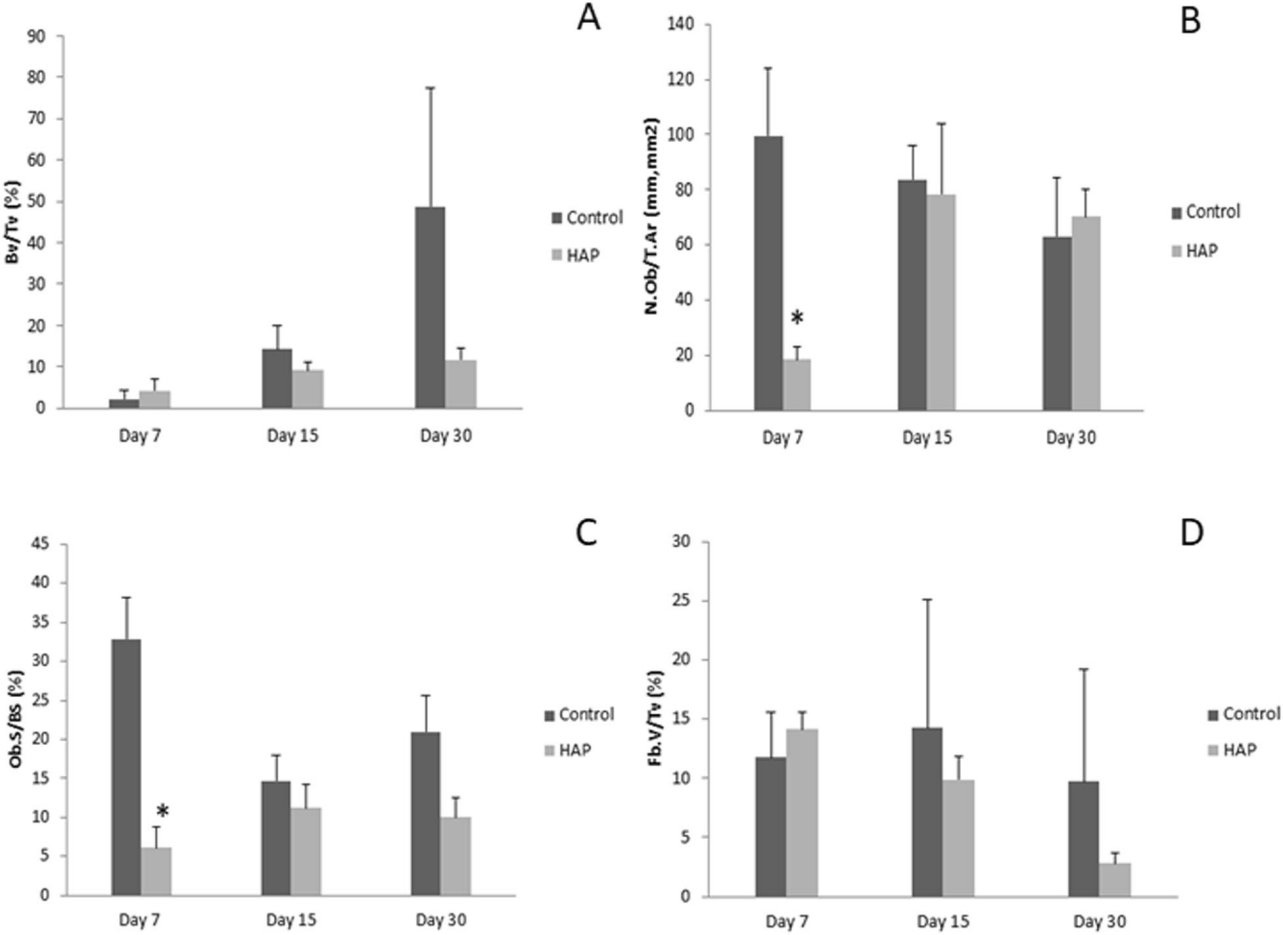
**A** Bone volume as a percentage of tissue volume (BV/TV, %) 7, 15 and 30 days after surgical procedure for control (no treatment) and hydroxyapatite group (bone defects filled with HAP particles). p > 0.05. **B** Number of osteoblasts per unit of tissue area (N.Ob/T.Ar) after 7, 15 and 30 days of the surgical procedure for control group (no treatment) and HAP groups (bone defects filled with HAP particles). *p < 0.05 vs. control group (One-way ANOVA with Tukey’s test). **C** Osteoblast surface as a percentage of bone surface (Ob.S/BS, %) after 7, 15 and 30 days of surgical procedures for control group (no treatment) and HAP group (bone defects filled with HAP particles). Statistical differences observed in day 7. *p < 0.05 vs. control group (One-way ANOVA with Tukey’s test). **D** Fibrosis volume as a percentage of tissue volume (Fb.V/TV, %) analyzed after 7, 15 and 30 days of surgical procedures for control group (no treatment) and HAP groups (bone defects filled with HAP particles). p > 0.05

Regarding bone volume, no significant differences (*p* > 0.05) were noticed between groups after 7, 15 or 30 days of HAP implantation. The results are demonstrated in Fig. 5A.

The number of osteoblasts inside the defect area (N.Ob/T.Ar) was lower in the HAP group (18.4 ± 4.1/mm^2^) when compared to the control group (99.7 ± 24.1/mm^2^) after 7 days post-implantation. No significant statistically differences (*p* > 0.05) were found after 15 and 30 days post-implantation (Fig. 5B).

In a similar way, the osteoblast surface as a percentage of the bone surface was higher in control group (32.8% ± 5.2%) when compared with HAP group (6.1% ± 2.6) after 7 days post-implantation. However, no significant statistically differences (*p* > 0.05) were observed after 15 and 30 days in the control or HAP groups (Fig. 5C).

Finally, the analysis of the percentage of the fibrotic tissue inside the bone defect revealed no significant statistically differences (*p* > 0.05) between groups for all periods evaluated in this study (Fig. 5D).

#### Immunohistochemistry for RUNX-2

For all groups, immunostaining was detected in the cytoplasm of cells inside the bone defect with brown color. In the control group, an intense immunostaining was noticed to cells in the granulation tissue after 7 days of HAP exposure. Following 15 days, the same pattern was found. After 30 days, a weak RUNX-2 immunoexpression was detected.

**Fig. 6:**
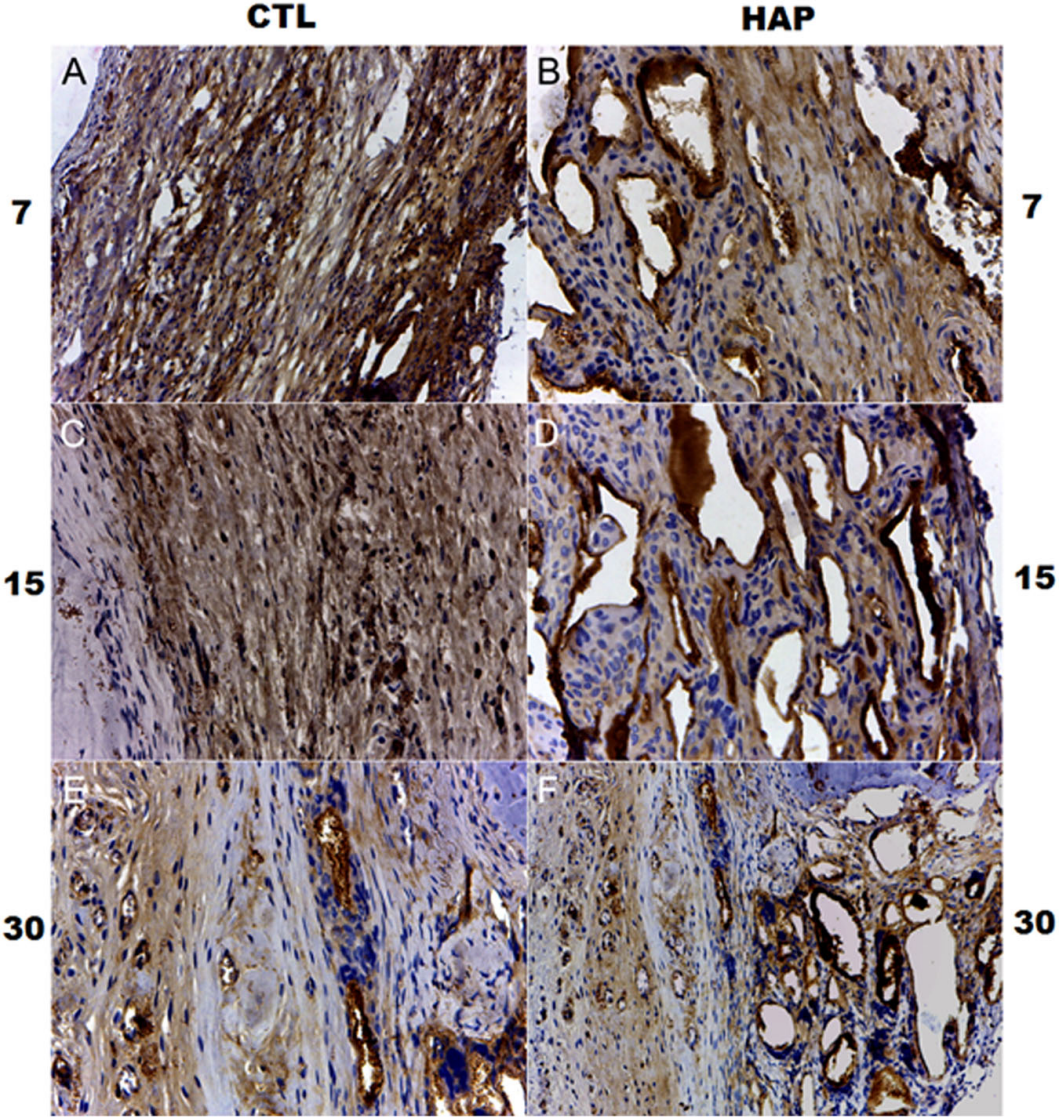
Immunohistochemistry of RUNX-2 in rat calvaria following HAP implantation after 7, 15 and 30 days post-implantation: CTRL control group, HAP hydroxyapatite group. ABC. stain. ×40 magnification

In the groups treated with HAP, interesting findings were observed. In the group treated with HAP for 7 days, a strong RUNX-2 immunoexpression was detected to cells in the granulation tissue as well as some cells in close contact with biomaterial. The same pattern was observed after 15 days of HAP exposure with, a strong immunostaining. After 30 days, a moderate immunostaining was detected. These results are shown in Fig. 6.

## Discussion

The use of various types of HAP has been widely described in the scientific literature. This material can be used, alone or in combination with collagen membranes, for bone reconstruction and other clinical applications [17]. An earlier study conducted by our research group, has originally designed HAP from fish waste. The results revealed no cytotoxic effects in multiples organs of rats such as, liver, kidney and lungs, associated with good biocompatibility in vivo [14]. The promising potential of HAP from fish waste is due to sustainable waste management and hazard reduction in terms of production and manufacturing, including bio-processes; enhancing company responsibility on products. Indeed, the approach plays an important role for human health and environment. In this study, we plan to dedicate further efforts to investigate HAP obtained from fish waste by means of cytotoxicity, osteogenesis and bone repair in vitro and in vivo.

First, the Alamar Blue assay showed no cytotoxic effects in osteogenic cell culture treated with HAP extract. Of particular importance was the significant reduction rate of Alamar Blue reagent after 3 and 6 days of conditioned cell culture, when compared to the control group. The data clearly indicates that cell viability may be improved by treatment with HAP extract. By comparison, Panda et al. [18] found an increase of cell viability with the exposition to HAP from fresh water fish (*Labeo rohita* and *Catla catla*) scales using mesenchymal stem cells in vitro. Boutinguiza et al. [19] found no cytotoxic effects of HAP extracted from swordfish and tuna fish bones, by MTT assay using mouse calvaria cells. These findings were also verified by others [20, 21]. Our results are fully in line with the aforementioned findings.

Moreover, our results demonstrated that DNA concentration was lower in HAP group after 6 days of exposure. This may indicate that cell proliferation rate was lower in this group. It is important to highlight that Alamar Blue reagent reduction was higher in the HAP-treated cells, showing that HAP from fish waste is biologically active. Venkatesan et al. [22] have assumed that mesenchymal stem cells exposed to HAP from salmon decreased cell proliferation viability in vitro. In the study of Piccirillo et al. [23], no cytotoxicity induced by HAP-based materials from Atlantic cod fish bones was found to osteosarcoma cell lineage. The same results were found by Pon-on et al. [24]. Fang et al. [21] have postulated high biocompatibility as a result of the ability to guide cell proliferation and migration in osteoblastic cell culture treated with HAP from fish (*Carassius auratus)* scales. Taken together, our results indicate that HAP from fish waste is not cytotoxic in vitro.

Regarding the expression of the osteogenic RUNX-2 and BMP4 genes, no remarkable differences were found to MC3T3-E1 pre-osteoblastic mouse cells cultivated or not in HAP-conditioned medium. These data suggest that HAP does not have osteoinductive properties in vitro, i.e., the biomaterial does not have the practical ability for differentiating precursor cells toward the osteogenic lineage and/or bone-forming activity. However, in vivo study demonstrated that HAP from fish waste was able to induce strong immunoexpression of Runx-2 for 7, 15 or 30 days post-implantation in this setting. Certainly, these discrepancies are due to differences into the experimental design. There is of course a broad consensus on this issue since in vitro studies does not consider the biological conditions that occurs in vivo. Even so, Hokmabad et al. [25] have revealed that incorporating HAP nanoparticles into nanofibers improve the expression of osteogenic marker genes (*Runx-2, Bglap, Bmp-2* and *Dspp*) in human dental pulp stem cells in vitro. These findings were confirmed by others [26–28]. Taken as a whole, we assume the HAP derived from fish is able to induce Runx-2 expression in bone cells in vivo.

In vivo analysis also demonstrated good biological response after HAP implantation as depicted by the presence of granulation tissue in an organized and orderly manner. These findings have been fully extended by histomorphometric parameters that showed no significant differences between HAP and control groups with respect to the percentage of bone formed (BV/TV %) as well as some cellular parameters evaluated in this study (N.Ob/T.Ar %, Ob.S/BS %), only except in day 7 for osteoblastic-related data. This difference may be due to the physical barrier that the biomaterial can create at the beginning of bone repair. Brum et al. [29] have assumed that HAP may have a lower degradation rate over other kinds of biomaterial. Certainly, this contributes to physical resistance of bone, but the presence of biomaterial could interfere with bone formation. In the study of Mondal et al. [20], *Labeo rohita* fish scales were chemically treated for obtaining HAP. Microscopic analysis pointed out bone formation on the HAP surface, which could indicate bioactivity and osteointegration after three months of HAP implantation [20].

## Conclusion

In summary, we conclude that HAP obtained from fish waste is a biocompatible material in vitro and in vivo. These findings were supported by previous studies (Table 1). However, further studies are still necessary, especially for the analysis to go on for a longer period of time, attempting to clarify the positive or negative findings. Certainly, HAP from fish waste is a promising possibility that should be explored more carefully by tissue-engineering or biotechnology.

**Table 1:** In vitro and in vivo studies of hydroxyapatite (HA) from fish in alphabetical order

Author(s)	Source of HA	Experimental design	Analysis	Main results
Boutinguiza et al. [19]	Sword fish (*Xiphias gladius*) and tuna (*Thunnus thynnus*)	In vitro: Mouse calvaria MC3T3-E1 cells	MTT assay	↑ cell viability
Fang et al. [21]	Fish (*Carassius auratus*) scales	In vitro: MC3T3-E1 osteoblastic cell culture	In vitro: cell number, attachment and morphology (confocal microscopy)	↑ proliferation rate ↑ cell migration
Mondal et al. [20]	Fish (*Labeo rohita*) scale	In vitro: RAW macrophage-like cell line In vivo: bone defects in Wistar rats	In vitro: MTT assay In vivo: histological analysis	↑ cell viability ↑ bone repair
Panda et al. [18]	Fresh water fish (*Labeo rohita* and *Catla catla*) scales	In vitro: Mesenchymal stem cells (MSCs)	MTT assay DNA quantification	↑ cell viability ↑ DNA content
Piccirilo et al. [23]	Atlantic cod fish bones	In vitro: culture with osteosarcoma cell line (Saos-2 cells) and f human bone marrow stromal cells (hBMSCs)	DNA quantification ALP analysis	No significant changes
Pon-On et al. [24]	Water fish (*P. jullieni*)	In vitro: rat osteoblast-like UMR-106 cells	MTT assay ALP analysis	↑ cell viability ↑ ALP activity
Venkatesan et al. [22]	Salmon (*Salmo salar*)	In vitro: mesenchymal stem cells (MSCs)	MTT assay	↓ cell viability ↓ cell growth
